# Alginate–Chitosan Gel Microbeads for PhiKZ Encapsulation as a Model of Bacteriophage Delivery to Combat *Pseudomonas aeruginosa*

**DOI:** 10.3390/gels12060544

**Published:** 2026-06-17

**Authors:** Liubov I. Popova, Elizaveta A. Akoulina, Evgeniia Yu. Parshina, Timofey A. Tarasov, Hejia Yue, Qing Peng, Ying Zhang, Andrei A. Dudun, Anton P. Bonartsev, Olga S. Sokolova, Tolbert Osire

**Affiliations:** 1 Biology Department, Shenzhen MSU-BIT University, 1 International University Park Road, Shenzhen 518172, China; popovaliubov@smbu.edu.cn (L.I.P.); sokolova@mail.bio.msu.ru (O.S.S.); 2 Faculty of Biology, M.V. Lomonosov Moscow State University, Leninskie Gory 1-12, 119234 Moscow, Russiaant_bonar@mail.ru (A.P.B.); 3 Department of Central Laboratory, The Second Affiliated Hospital, School of Medicine, The Chinese University of Hong Kong, Shenzhen & Longgang District People’s Hospital of Shenzhen, Shenzhen 518172, China; pengqing@cuhk.edu.cn (Q.P.); geeyoser@stu2025.jnu.edu.cn (Y.Z.); 4 Research Center of Biotechnology of the Russian Academy of Sciences, Leninsky Ave., 33, Bld. 2, 119071 Moscow, Russia; dudunandrey@mail.ru

**Keywords:** bacteriophage, biopolymer gel microbeads, antibacterial materials, *Pseudomonas aeruginosa*

## Abstract

Wound infections due to antibiotic resistance pose a global public health problem. Phage therapy is a promising approach to address this issue. To improve localization, phage stability, delivery, and antibacterial performance, we propose polymer mix gel microbeads encapsulated with phages as a model for the delivery of phiKZ bacteriophage to combat *Pseudomonas aeruginosa*. Phages were loaded into the alginate pre-gel under magnetic stirring, with further cross-linking by chitosan and/or Ca^2+^ ions. The obtained gel microbeads were characterized using FTIR and Raman spectroscopy, and their cytotoxicity and antimicrobial properties were evaluated. This study demonstrated the efficient loading of high-titer phage lysate, achieving up to 99% encapsulation efficiency for alginate–chitosan microbeads. The key characteristics of the microbeads include stable physicochemical properties, slow but continuous phage release over 48 h in physiological saline, and low cytotoxicity. The phage-loaded microbeads demonstrated strong in vitro antimicrobial activity against *P. aeruginosa* PAO1, resulting in mean reductions of 6.9 log_10_ and 4.8 log_10_ CFU/mL for alginate and alginate–chitosan formulations, respectively. This corresponded to a decrease in bacterial concentration from approximately 1.1 × 10^11^ CFU/mL in untreated controls to 1.1 × 10^5^ CFU/mL and 7.7 × 10^6^ CFU/mL for alginate and alginate–chitosan formulations after 3 h of incubation.

## 1. Introduction

The global incidence of tissue/bone injuries, as well as chronic wounds, has increased spontaneously in recent decades, often as a result of age, obesity, diabetes, or cardiovascular disease [1,2]. Coincidentally, recent reports from Lancet have indicated that bacterial infections, specifically those resulting from the emergence of antimicrobial-resistant strains (superbugs)—most notably *Pseudomonas aeruginosa*, *Escherichia coli*, *Salmonella aureus*, *Klebsiella pneumoniae*, *Streptococcus pneumoniae*, and *Acinetobacter baumannii*—pose a major challenge for global public health, leading to extensive inpatient hospitalization, a higher risk of complications, a substantial cause of morbidity/mortality, and increased costs of medical treatment, thereby exerting significant pressure on healthcare systems and public health [3,4,5].

The extensive overuse of antibiotics in healthcare, agriculture, and livestock production has aggravated the antimicrobial resistance challenge, with recent predictions suggesting an estimated 35 million fatalities by 2050 directly associated with antimicrobial-resistant strains [6]. This has sparked interest in exploring alternative antimicrobials that are efficient against superbugs, including the design of improved functionalized biomaterials that offer stability and controlled release of the antimicrobial agents to infected tissues, offering significant benefits compared to traditional approaches [7,8].

Phage therapy is one of the cutting-edge approaches to combat multidrug-resistant infections. However, although existing studies indicate the promise of phage therapy, the role of formulation strategies in improving phage stability, delivery, and antibacterial performance still requires further optimization; current methods often suffer from issues such as low phage release, instability under physiological conditions, and inconsistent encapsulation efficiency [8]. Additionally, although recent studies have explored encapsulating or immobilizing phages within biocompatible hydrogel polymers, there is still insufficient research on this approach and the application of these formulations as alternative antibacterial compositions. The pursuit for innovative biomaterials that integrate functionality, sustainability, and biocompatibility has placed natural polymers such as alginate and chitosan at the forefront of biomedical innovation [9]. Chitosan’s structural similarity to extracellular glycosaminoglycans promotes cellular adhesion and integration, while alginate’s moderate gelling properties (via divalent cations like Ca^2+^) enable safe cell encapsulation [10]. Their biodegradability through enzymatic or hydrolytic mechanisms guarantees progressive elimination from the body, hence diminishing long-term implantation dangers. The polyelectrolytic nature of chitosan (cationic) and alginate (anionic) is complementary, enabling robust self-assembly via electrostatic interactions into stable complexes such as nanoparticles, hydrogels, microbeads, etc. These polymers further exhibit intrinsic bioactivities, as chitosan possesses the ability to disrupt microbial membranes through cationic interactions with teichoic acids, thus ensuring its broad antibacterial properties. Meanwhile, alginate effectively regulates wound exudates due to its fluid-absorbing properties, hence sustaining a moist milieu in wounds, which promotes regeneration [11,12].

In our study, in addition to seaweed alginate, we used bacterial alginate obtained by biosynthesis by *Azotobacter vinelandii* [13]. Bacterial alginate differs from seaweed alginate in its monomeric composition of M/G and is usually acetylated [14]. This allows for the first-time use of a material produced by bacteria to encapsulate bacteriophages used to fight infectious bacteria, just as we previously used it to encapsulate antimicrobial peptides [15].

Several phage-integrated polymer-based biomaterials have been developed for broad applications, such as drug delivery, controlled release, and bacterial infection management [16]. For example, Pinto et al. presented a comprehensive review of novel delivery systems for bacteriophage applications in targeted antibacterial control and chronic wound treatment [17]. To achieve controlled phage release, Moghtader et al. developed sodium alginate–gelatin microbeads coated with chitosan via dehydrothermal cross-linking. While the gel matrices exhibited some phage instability compared to lyophilized microbeads, they achieved approximately 80% release of loaded T4 phages over 24 h [18]. Chen et al. developed a two-phage cocktail and antibiotic-loaded emulsion-based hydrogel system (with 10^10^–10^11^ PFU/mL FJK.R9-30 phage load) also containing alginate microbeads (with 10^7^ PFU/mL MK.R3-15 phage load) and achieved significant phage and antibiotic meropenem activity and controlled release for at least a week [19]. Based on the literature, current phage delivery systems often suffer from rapid initial release, inconsistent antibacterial activity, and limited standardization owing to variability in material production protocols and characterization methods. Therefore, optimizing high-titer phage production, phage integration, and biomaterial composition may offer a novel therapeutic solution for chronic, drug-resistant wound infections.

This study aims to establish a robust and reproducible phage encapsulation protocol for alginate–chitosan gel microbeads, compared to pure alginate microbeads as a control, to ensure stable phiKZ bacteriophage loading capacity with prolonged release properties for personalized medicine and antimicrobial applications, serving as a potential delivery system and targeted treatment platform for drug-resistant *P. aeruginosa* in infected wounds.

## 2. Results and Discussion

### 2.1. Gel Microbead Production

Gel microbeads were produced based on seaweed (sALG) or bacterial (bALG) alginate with or without chitosan (CS), including encapsulation of phiKZ bacteriophage to exert antimicrobial activity against *P. aeruginosa* PAO1. As a result, eight types of microbeads were obtained—bAN, sAN as blank bacterial and seaweed alginate microbeads, bAP and sAP as alginate microbeads with phage encapsulation, bANCS, sANCS as alginate–chitosan blank microbeads, bAPCS, and sAPCS—with corresponding phage encapsulation (abbreviation decoding: bA—bacterial alginate, sA—seaweed alginate, N—no phage, P—phage-loaded, CS—chitosan; Table 1).

The initial phage load from the high-titer lysate for both alginate and alginate–chitosan microbeads was 4.3 × 10^12^ PFU/mL. Due to the high biopolymer content in alginate–chitosan beads, the dried mass of these beads was approximately twice that of alginate only (0.008 ± 0.001 g compared to 0.004 ± 0.001 g). Despite this difference, the final phage content after all washing steps was comparable, reaching approximately 10^14^ plaque forming units (PFU)/mg of dried beads (Table 2). A notable difference was observed among the bead types in terms of phage encapsulation efficiency. The alginate–chitosan formulations (sAPCS and bAPCS) achieved nearly 100% phage-loading efficiency, whereas the pure alginate beads (sAP and bAP) demonstrated lower efficiencies of approximately 40% and 50%, respectively. This observation is consistent with previous studies on alginate–chitosan microbeads used for phage encapsulation [20]. Enhanced encapsulation is primarily attributed to the formation of a polyelectrolyte complex between the cationic amino groups of chitosan and the negatively charged capsid and tail fibers of bacteriophages [21,22].

**Figure 1:**
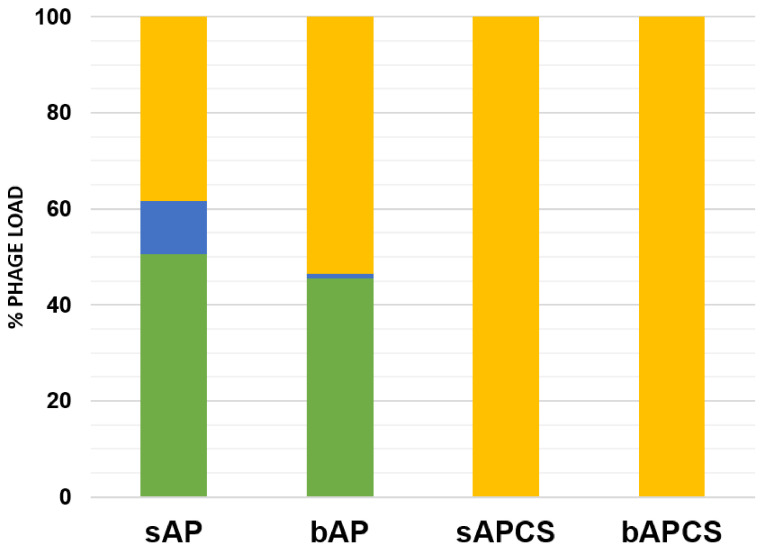
Phage load distribution for different bead types is expressed as a percentage of the initial phage input. Bars represent phage losses during processing of alginate (sAP and bAP) and alginate–chitosan (sAPCS and bAPCS) formulations (according to Table 1): green—loss after washing step 1, blue—loss after washing step 2, yellow—amount of encapsulated phage.

Notably, the seaweed-derived alginate formulations exhibited lower phage-loading efficiency and less efficient release kinetics compared to the bacterial alginate. Studies have shown that the physicochemical properties of alginate significantly influence the performance of encapsulated phage delivery systems [21], which could be partly associated with the varying molecular weight and composition of alginate, depending on the source. Typically, seaweed-derived alginate contains higher proportions of guluronic acid blocks, leading to stronger ionic cross-linking and stiffer gels, whereas bacterial alginate characteristics can be controlled, such as different molecular weights with different monomer compositions resulting in altered gel elasticity and porosity [13,23]. These structural differences can impact the encapsulation efficiency and phage diffusion inside the material, demonstrating an approximately 10% difference in phiKZ load into alginate-based beads (Figure 1).

### 2.2. Microbead Characterization

**Figure 2:**
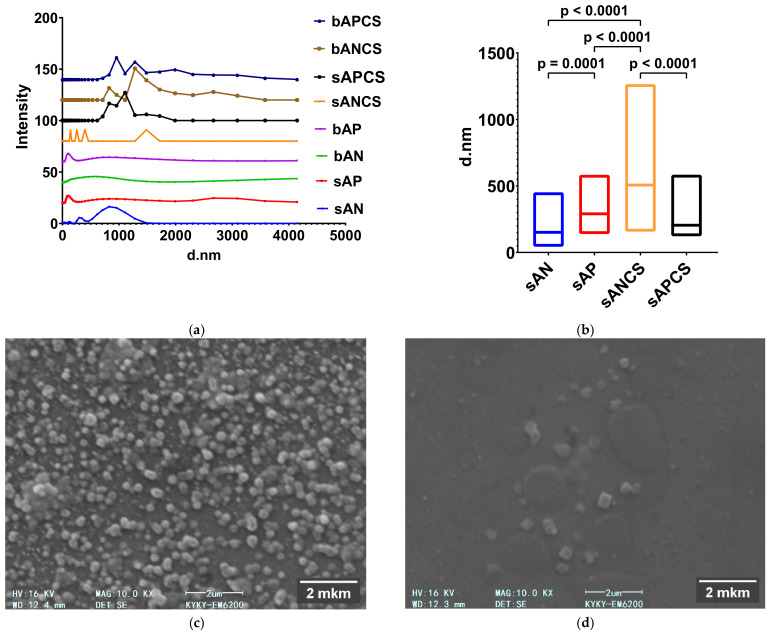

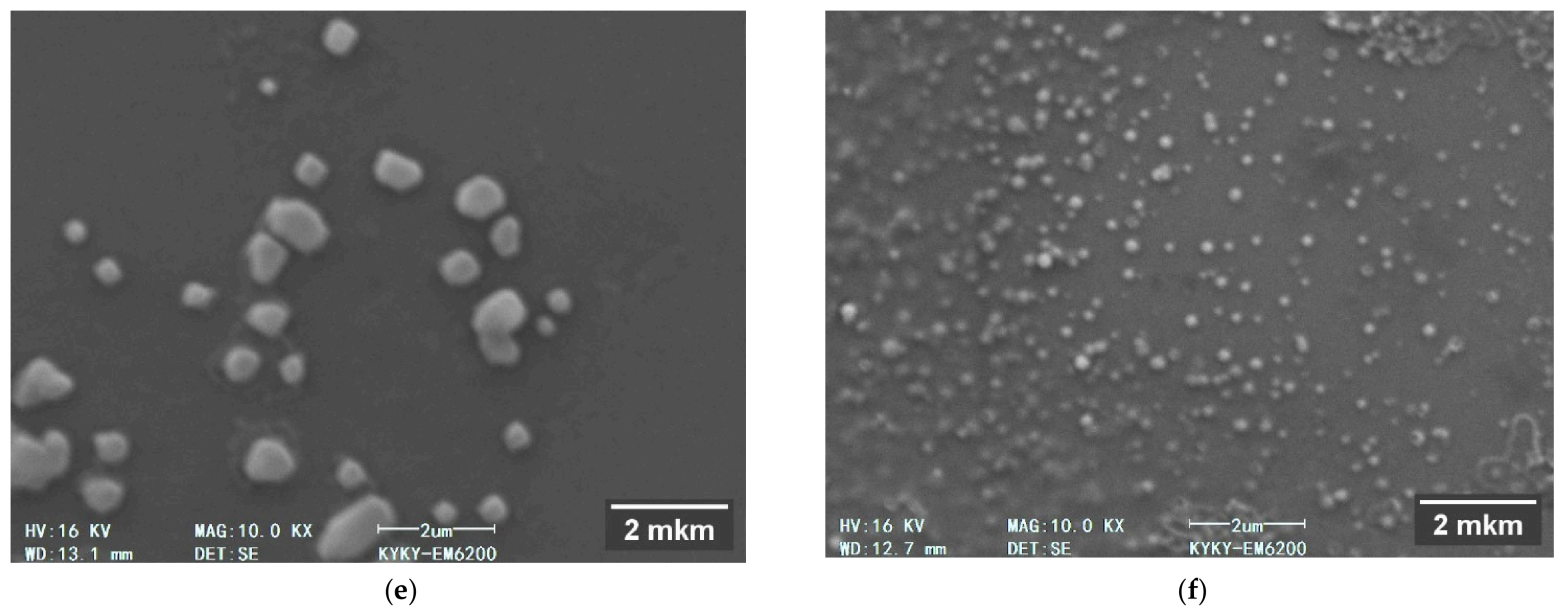
Microbead size distribution by intensity ((**a**), *n* = 9); size distribution based on diameter measurements on SEM photos (**b**), sAN (*n* = 200), sAP (*n* = 19), sANCS (*n* = 56), sAPCS (*n* = 195), one-way ANOVA followed by Tukey’s post hoc multiple comparison test; SEM of alginate-based beads without and with phage—sAN (**c**) and sAP (**d**), alginate–chitosan beads without and with phage—sANCS (**e**) and sAPCS (**f**) (according to Table 1).

Mean sizes (Figure 2a) by Polydispersity Index (PDI) measurement of the blank and phage-loaded gel microbeads were assessed by dynamic light scattering in physiological saline. The results showed no significant difference between seaweed and bacterial alginate used as the base material for microbeads. After the addition of phages in the system, we observed a more loosened microbead arrangement. Based on the SEM images, the alginate–chitosan sANCS microbeads possessed a large fraction of beads (Figure 2e), while the phage-incorporated alginate–chitosan sAPCS microbeads (Figure 2f) were smaller in size, comparatively similar to that of alginate-based sAN and sAP microbeads (Figure 2c,d). We observed that the addition of chitosan to the polymer mixture significantly increased the size of the microbeads, possibly due to the ionic interactions between the negatively charged alginate sites and positively charged chitosan [24]. However, the subsequent addition of phiKZ to the polymer system gradually decreased the microbead size. The same effect was observed with the addition of endolysins to chitosan–alginate microparticles [8].

Additionally, the ζ-potentials of the microbeads were measured using a Litesizer DLS 700. Alginate-based microbeads exhibited (Table 3) highly negative ζ-potentials, both with and without incorporated phages, measuring approximately −36.6 ± 3.3 mV for sAN and −33.1 ± 2.7 mV for sAP, suggesting altered surface charge characteristics consistent with phage association at or near the microbead surface [25]. In contrast, alginate–chitosan microbeads (sANCS) exhibited a slightly positive ζ-potential of +4.3 ± 1.5 mV, consistent with partial chitosan surface coverage. Following phage incorporation, the ζ-potential of alginate–chitosan microbeads shifted markedly toward negative values, reaching −18.7 ± 1.3 mV for sAPCS, indicating substantial modification of the surface charge properties. Previous studies have shown that alginate and chitosan form electrostatically mediated polyelectrolyte complexes and that the charge ratio of alginate/chitosan strongly influences the size of the complex, its compactness, ζ-potential, encapsulation efficiency, and release behavior [26,27,28,29]. In addition, the gelation of Ca-alginate is well known as an ionotropic cross-linking process involving negatively charged carboxylate groups of alginate and Ca^2+^ ions, providing a physical capture network [30]. It is known that bacteriophages and viruses behave as pH-dependent charged colloids, and their electrostatic charge determines their colloidal behavior and sorption processes [31]. In particular, for bacteriophages, it has been shown that cationic compounds, including chitosan, interact with negatively charged phage particles through electrostatic interactions, and this interaction is influenced by pH and ionic strength [32], which explains the decrease of the ζ-potential for APCS. Moreover, chitosan-based matrices have been reported to immobilize bacteriophages through the electrostatic association between positively charged chitosan fragments and negatively charged phage capsids [33]. Thus, the increase in encapsulation efficiency (Figure 1, Table 2) is most plausibly explained by the cumulative contribution of physical capture to the Ca-alginate network, which reduces leakage due to complexation mediated by chitosan and the electrostatic association between negatively charged phiKZ particles and protonated domains, rich in chitosan.

In the absence of phages, chitosan can partially neutralize negatively charged alginate domains and promote aggregation/bridge-like association between particles; such aggregation is especially expected near electrical neutrality, where alginate–chitosan polyelectrolyte complexes exhibit an increased apparent particle size [34]. In contrast, phiKZ behaves like a strongly negatively charged biocolloid: electrokinetic measurements showed a hydrodynamic diameter of approximately 156.9 nm and a ζ-potential of −48.2 ± 4.8 mV [35]. Consequently, phiKZ can provide additional anionic binding sites for protonated chitosan chains, reducing the number of free chitosan cationic sites, suppressing the formation of chitosan-mediated bridges, and shifting the charge of the bead surface towards more negative values. This interpretation is also consistent with previous studies showing that cationic compounds, including chitosan, interact with negatively charged bacteriophages through electrostatic interactions [32] and that chitosan-based matrices can immobilize phages through electrostatic associations between positively charged chitosan fragments and negatively charged phage capsids [33]. In our system, this mechanism was confirmed by a shift in the ζ-potential from +4.29 mV for sANCS to −18.67 mV for sAPCS. The observed decrease in size is most likely due to a decrease in aggregation and partial compaction/reorganization of the alginate–chitosan–phage interfacial layer rather than chemical degradation of the polymer matrix.

### 2.3. Microbead Composition Detected by Spectroscopy Analysis

**Figure 3:**
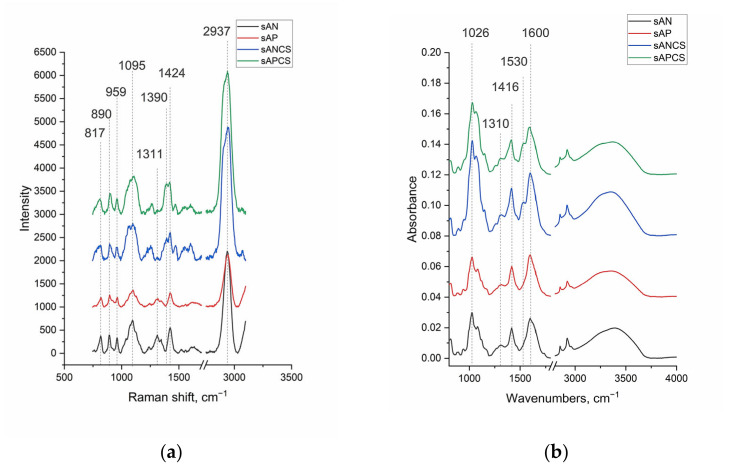
Raman (**a**) and FTIR (**b**) averaged spectra of seaweed alginate microbeads. Each subsequent spectrum has an offset along the y-axis to avoid overlapping.

The composition of gel microbeads based on seaweed-derived alginate was characterized using Raman and FTIR spectroscopy. According to Figure 3a, the Raman spectra of sAN and sAP correspond to the spectra of alginate. Some bands in the spectrum (817 cm^−1^ (C–C str), 959 cm^−1^ (C–O str)) match the positions characteristic of calcium alginate, while others (1095 cm^−1^ glycosidic ring breathing, C–C str, 1424 cm^−1^ COO− symmetric str) occupy intermediate positions between the bands typical of sodium alginate and calcium alginate [36,37]. This may indicate that the sample is a mixture of sodium alginate and calcium alginate.

**Figure 4:**
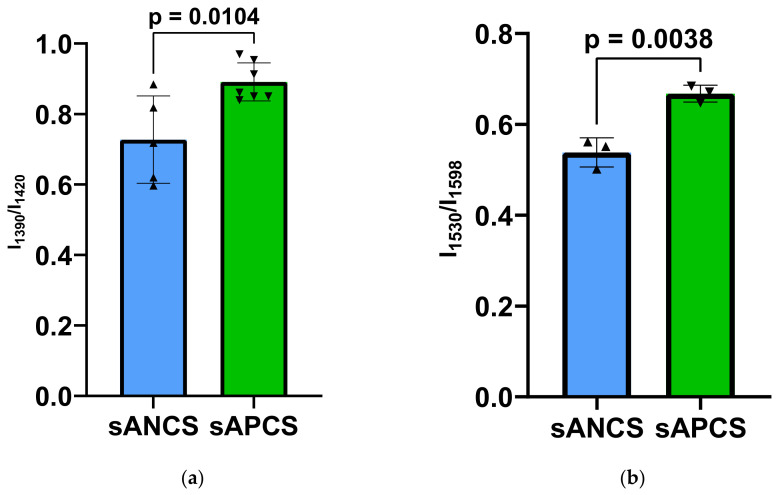
Relative amount of chitosan in microbead samples by Raman (**a**) and FTIR (**b**) spectra (*n* = 5 for (**a**) and *n* = 3 for (**b**), Student’s *t*-test).

The spectra of the sANCS and sAPCS samples differed from that of the alginate spectrum due to the presence of additional bands characteristic of chitosan. The presence of phages alters the spectral shape in the region of the 1390 cm^−1^ band (CH_2_ def, str(C–N), str(C–H), str(C–OH)) [38,39,40], which is characteristic of chitosan molecules. The ratio of the intensities of 1390 cm^−1^ and 1424 cm^−1^ bands (I_1390_/I_1424_) can be used to estimate the relative amount of chitosan in the alginate–chitosan mixture. It can be observed that the relative amount of chitosan increased upon phage addition (significant differences according to Student’s *t*-test, *p* < 0.05) (Figure 4).

Figure 3b presents the ATR-FTIR spectra of the microbeads. Similar to the Raman spectra, the spectra of the sAN and sAP samples correspond to those of alginate (1026 cm^−1^ C–C str, 1416 cm^−1^ COO− symmetric str, 1600 cm^−1^ COO− asymmetric str [36]). The spectra of samples containing chitosan exhibited additional bands (1530 cm^−1^ NH_2_ def, N–H str [38,40,41]). The ratio of the intensities of 1530 cm^−1^ and 1598 cm^−1^ bands (I_1530_/I_1598_) indicates an increase in the chitosan content in the sAPCS sample compared to that in sANCS (Figure 4), which corroborates the data obtained by Raman spectroscopy. However, when phages are added, the comparative spectra for sANCS and sAPCS indicated an increase in chitosan content, which could be related to the presence of negatively charged phage particles that probably provided additional sites for interaction with chitosan [33].

### 2.4. Microbead Antimicrobial Activity

In vitro antimicrobial activity was assessed by evaluating the inhibition of bacterial growth in broth culture and the subsequent determination of the reduction in colony forming unit (CFU) counts after 3, 6, and 9 h of incubation (Figure S1). Both sALG and bALG alginate microbeads were tested with and without chitosan coating and compared to blank (control) beads that lacked bacteriophage.

**Figure 5:**
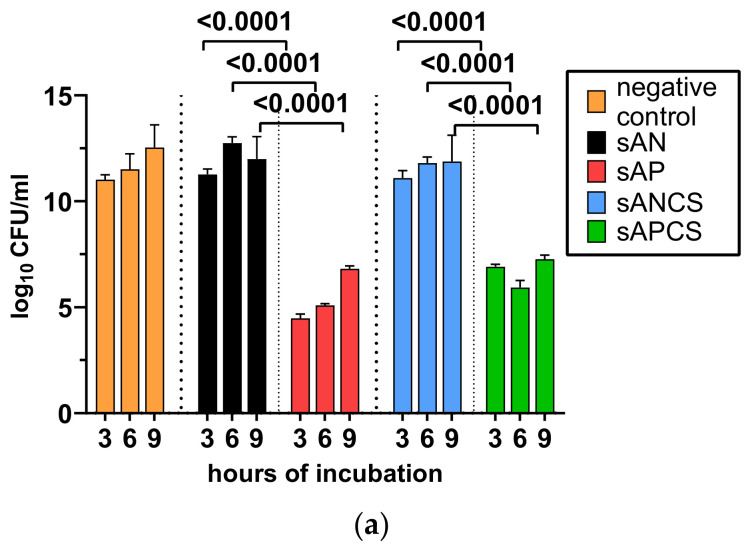

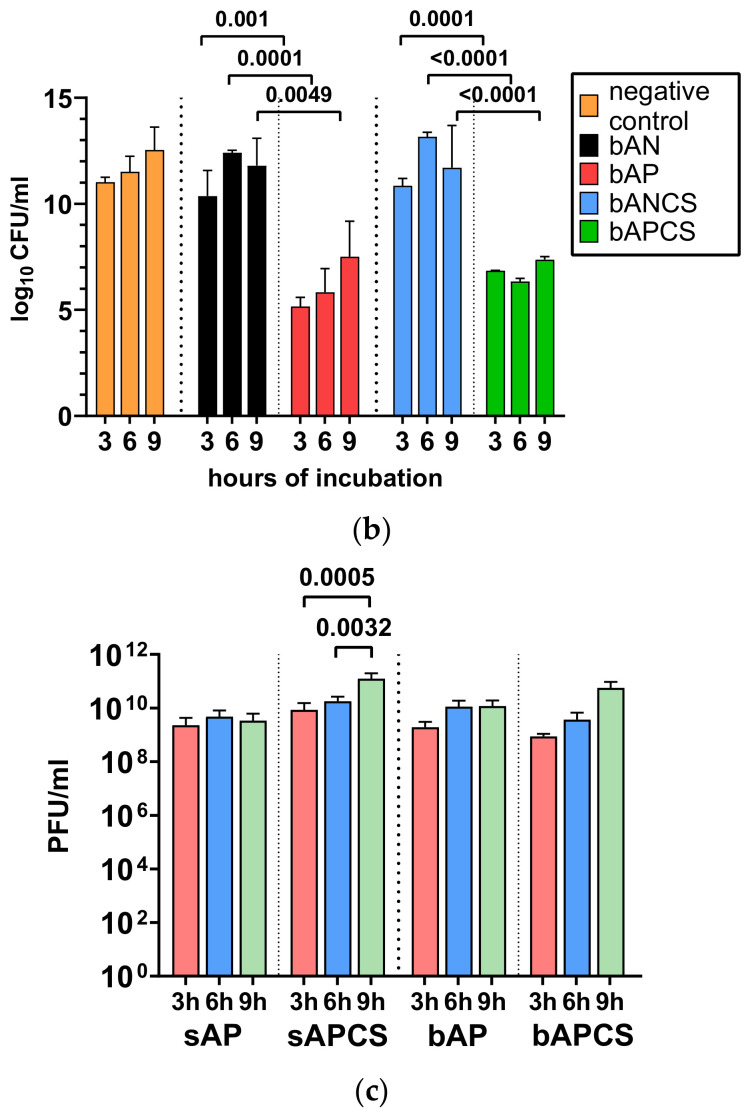
In vitro antibacterial activity of alginate and alginate–chitosan microbeads. Quantitative assessment of *P. aeruginosa* CFU/mL counts following treatment with microbeads based on seaweed alginate (**a**) and bacterial alginate formulations (**b**). Orange—negative control (only *P. aeruginosa* culture), black—alginate beads (bAN or sAN), red—alginate beads loaded with phages (bAP or sAP), blue—alginate–chitosan beads (bANCS or sANCS), green—alginate–chitosan beads loaded with phages (bAPCS or sAPCS); abbreviations used according to Table 1; *n* = 3 (one-way ANOVA followed by Tukey’s post hoc multiple comparison test). (**c**) In vitro evaluation of phiKZ bacteriophage propagation dynamics during a *P. aeruginosa* treatment with different types of microbeads (*n* = 3). Each set of three bars corresponds to a specific microbead type listed in Table 1. Red—3 h of incubation, blue—6 h of incubation, green—9 h of incubation. (lognormal Welch’s *t*-test).

Based on our results (Figure 5a,b, Table S1), all phage-loaded microbeads exhibited significant bactericidal activity compared with empty control beads starting from 3 h of incubation (*p* < 0.0001 for bAP and *p* = 0.0002 for bAPCS and *p* < 0.0001 for sAP and sAPCS, one-way ANOVA followed by Tukey’s post hoc multiple comparison test). After 3 h, sAP beads achieved a 7.2 ± 0.2 log_10_ CFU/mL reduction, whereas bAP beads reached a 6.5 ± 0.2 log_10_ CFU/mL reduction. Chitosan-coated beads (sAPCS and bAPCS) exhibited similar antibacterial performance regardless of alginate source, with an approximately 4.8 ± 0.1 log_10_ CFU/mL reduction after 3 h. Compared with alginate-based gel beads, the lower early antibacterial activity of chitosan-coated formulations may reflect slower phage diffusion associated with the chitosan domain’s possible barrier, which could affect the initial release of phages into the surrounding medium. Prolonged incubation up to 9 h maintained bacterial killing, with sAP and bAP beads showing a mean reduction of 4.5 ± 0.2 log_10_ CFU/mL (gradually decreased compared to 3h), while sAPCS and bAPCS beads demonstrated a mean reduction of 4.4 ± 0.3 log_10_ CFU/mL, indicating continued phage-mediated bacterial suppression. This observation may be related to the lower phage availability from alginate–chitosan microbeads, as also supported by prolonged phage activity observed in the *P. aeruginosa* infection model (Figure 5c).

The antibacterial activity observed in the present study is comparable to previously reported phage-loaded polysaccharide delivery systems, although direct comparison is limited by differences in bacterial host, phage type, formulation composition, and experimental conditions. For example, alginate–chitosan microparticles encapsulating bacteriophages ZCEC5 have been reported to achieve an approximately 4.5–5 log_10_ reduction of *E. coli* CFU after 10 h [20], while another alginate–chitosan-based system reduced *E. coli* O157:H7 by approximately 2.5 log_10_ CFU after 5 h under simulated intestinal conditions [42]. Similarly, chitosan nanoparticles with 97% encapsulation efficiency of phage HK6 against *Enterobacter cloacae* produced an approximately 4 log_10_ CFU reduction 2 h post-exposure [43].

PhiKZ phage propagation in vitro dynamics during the *P. aeruginosa* infection model (Figure 5c) were quantified in parallel with the antibacterial assays. For sAP and bAP beads, there were no statistically significant differences in phage titers between time points, with a release of 7.2 × 10^9^ PFU/mL (mean value) observed at 9 h. In contrast, sAPCS beads exhibited a statistically significant different phage release profile, with a phage titer of 8.4 × 10^9^ PFU/mL after 3 h to approximately 1.2 × 10^11^ PFU/mL at 9 h (*p* = 0.0005, Welch’s *t*-test). Notably, even after nine hours, all four phage-encapsulated microbead types maintained a detectable antibacterial effect (mean 4log_10_ CFU reduction), demonstrating that encapsulation supported prolonged phage activity over this time period. These findings underscore the dual role of chitosan in microbead composition: improving encapsulation efficiency through electrostatic interactions and modulating release kinetics by altering the microbead polymeric microstructure [20,21].

It is important to note, however, that the antibacterial activity of polymer microbead systems is influenced not only by the carrier composition but also by the specific properties of the phages used, including their host range, lytic activity, and physicochemical stability under encapsulation [22,33].

### 2.5. Phage Release in Physiological Saline Solution

**Figure 6:**
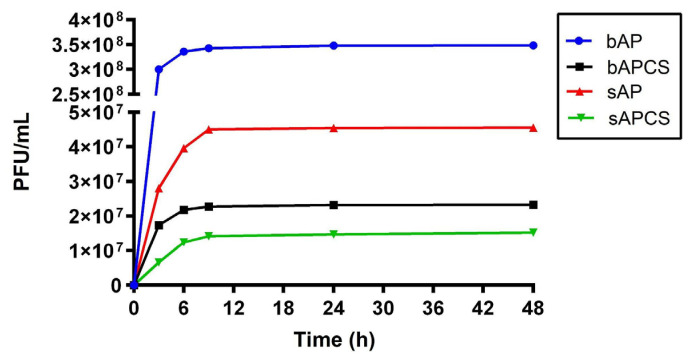
Release of phiKZ bacteriophage from alginate and alginate–chitosan microbeads in physiological saline solution during 48 h (*n* = 3, represented as mean).

The in vitro release dynamics of phiKZ phage from alginate-based gel microbeads were evaluated in physiological saline solution under shaking conditions at 37 °C (Figure 6). The majority of detectable phage release occurred during the initial incubation period, followed by relatively stable phage titers between 9 and 48 h for all formulations. Comparatively, the cumulative phage titer from the initial release, particularly from alginate–chitosan microbeads bAPCS and sAPCS (1.74 × 10^7^ and 6.6 × 10^6^ as mean PFU/mL, respectively) within the same time interval of 3 h, was lower than that for microbeads without chitosan (3.01 × 10^8^ and 2.82 × 10^7^ as mean PFU/mL for bAP and sAP, respectively), indicating a relatively slowed release by the former microbeads, followed by a slow phase of sustained release between 6 and 9 h (Figure 6). These differences are likely associated with the formation of a denser alginate–chitosan polyelectrolyte matrix that restricts phage diffusion and promotes stronger retention of negatively charged phage particles within the microbead structure in pH-neutral buffers, as also discussed by Rotman et al. Additionally, another kinetic of release from alginate beads could be explained by the swelling/degradation mechanism of calcium alginate hydrogel [22].

The observed release behavior correlated with the antibacterial activity profiles of the formulations. Alginate-only beads demonstrated stronger early antibacterial activity, likely due to greater initial phage availability, whereas alginate–chitosan formulations exhibited reduced early bacterial killing but more moderated phage diffusion behavior (Figure 5a,b).

The subsequent gradual release is suitable for the prevention of bacterial infections, in agreement with a previous study by Gomaa and co-authors [44]. Moghtader et al. also designed alginate–chitosan microbeads that achieved complete phage release in 24 h [18]. The microbeads in the current study, however, achieved small and slow but continuous phage release in physiological saline solution for at least 48 h (Figure 6). Our findings suggest that alginate and alginate–chitosan beads exhibit distinct functional release characteristics that may be advantageous for different therapeutic applications.

### 2.6. Cytotoxicity

**Figure 7:**
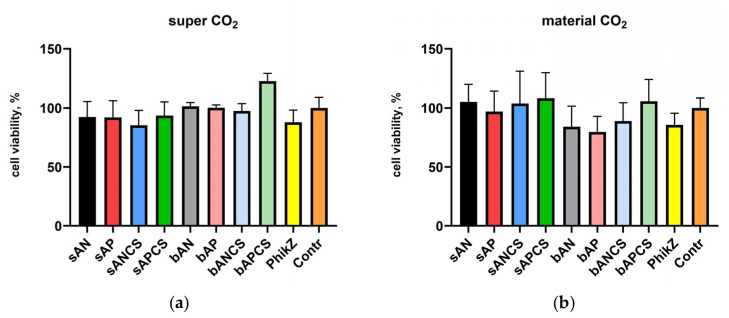
Microbeads (**b**) and their supernatant after 24 h incubation (**a**) cytotoxicity under normal conditions. (*n* = 10, all comparisons were non-significant by one-way ANOVA followed by Tukey’s post hoc multiple comparison test).

All types of microbeads were tested for their cytotoxicity effect on the 3T3 fibroblast cell line with pure phages as an additional control. The supernatant of 200 μL of materials was incubated in 800 μL of growth media for 24 h, and the remaining material was resuspended in 1000 μL of fresh growth media and tested separately. Cytotoxicity was tested in normal conditions with 6% CO_2_ and 37 °C, and in incubators without the CO_2_ control (0.03% CO_2_, 37 °C) (Figure S2). In normal conditions with 6% CO_2_, there is no statistical difference between all groups and the control group (Figure 7). In conditions without CO_2_, cells in the control group were only 2% of the control cells with 6% CO_2_.

Although there are no studies on the cytotoxicity of alginate–chitosan mix with bacteriophages, it has been shown previously that blank alginate–chitosan hydrogels do not have a cytotoxicity effect on fibroblast cells and even provide good support for cell proliferation [10]. In our previous studies, we also showed that blank bacterial, as well as seaweed-derived alginate, also has no cytotoxic effect on eukaryotic cells [45]. There are no studies on the cytotoxicity of pure phiKZ or its use in the form of any encapsulated phage cocktail. However, other studies have shown that chitosan compositions with encapsulated bacteriophages [46] and hydrogels with bacteriophages [47] do not have cytotoxic effects on eukaryotic cells, which is in accordance with the results of this study, indicating that under normal conditions, the addition of phiKZ bacteriophages at a concentration of 4.3 × 10^12^ PFU/mL does not affect eukaryotic cell proliferation during 24 h.

## 3. Conclusions

This study presents the successful development of alginate-based gel microbeads encapsulating the phiKZ bacteriophage, serving as a model system for potential phage delivery to combat *Pseudomonas aeruginosa* infections. Spectroscopic analyses (FTIR, Raman spectroscopy, particle size analysis, and ζ-potential measurements) confirmed the encapsulation of PhiKZ into alginate and alginate–chitosan microbeads. The developed formulations showed efficient phage loading, with encapsulation efficiency reaching approximately 40–50% for alginate-only microbeads and nearly 99% for alginate–chitosan microbeads. The ζ-potential measurements further supported phage incorporation and surface-associated charge changes, with alginate-based formulations showing strongly negative values (−36.6 ± 3.3 mV for sAN and −33.1 ± 2.7 mV for sAP) and alginate–chitosan formulations displaying a shift in surface charge after phage loading (+4.3 ± 1.5 mV for sANCS and −18.7 ± 1.3 mV for sAPCS). Particle-size analysis also indicated changes in bead diameter after phiKZ addition, suggesting interactions between phage particles and the polymer matrix during gel formation.

In vitro release studies demonstrated that both alginate and alginate–chitosan formulations released detectable levels of active phiKZ particles over 48 h in physiological saline. In the antibacterial assay, phage-loaded alginate and alginate–chitosan microbeads produced mean reductions of 6.9 log_10_ CFU/mL and 4.8 log_10_ CFU/mL, respectively, against *P. aeruginosa* PAO1 after 3 h of incubation. These values corresponded to a decrease in bacterial counts from approximately 1.1 × 10^11^ CFU/mL in untreated controls to 1.1 × 10^5^ CFU/mL for alginate beads and 7.7 × 10^6^ CFU/mL for alginate–chitosan beads. The formulations also demonstrated low cytotoxicity under the tested conditions.

The contribution of this study is the comparative evaluation of pure alginate and alginate–chitosan microbeads as functionally distinct phage-delivery systems. Rather than identifying one formulation as more efficient for phiKZ encapsulation, the results show that phiKZ-loaded pure alginate beads provide stronger early antibacterial activity within 3 h, while alginate–chitosan beads modulate and prolong the release behavior. This suggests that the two systems may be suitable for different therapeutic scenarios or may be combined in future formulations to integrate rapid initial bacterial reduction with a prolonged phage availability.

Overall, this study provides a simple and reproducible approach for developing PhiKZ-loaded alginate-based microbeads with preserved phage infectivity, measurable release, strong in vitro antibacterial activity, and low cytotoxicity. Future work will focus on optimizing phage loading in alginate-only beads, testing combined alginate/alginate–chitosan bead formulations, evaluating interactions with eukaryotic wound-relevant cell cultures, and validating antibacterial efficacy and phage-release behavior in more complex in vitro and in vivo infection models.

## 4. Materials and Methods

### 4.1. Materials

The chitosan (purity ≥ 95%, MW = 200,000) was purchased from Macklin, Shanghai, China, Lot no. C17985865, CAS: 9012-76-4. The components of the Burk’s culture medium: dipotassium phosphate (K_2_HPO_4_·3H_2_O), magnesium sulfate (MgSO_4_·7H_2_O), sodium chloride (NaCl), sodium molybdate (Na_2_MoO_4_·2H_2_O), calcium carbonate (CaCO_3_), iron (II) sulfate (FeSO_4_·7H_2_O), sodium citrate, calcium chloride (CaCl_2_), monopotassium phosphate (KH_2_PO_4_), sucrose, agar. All reagents were of analytical grade (purity ≥ 99%) and were obtained from Merck (former Sigma-Aldrich, Darmstadt, Germany). When isolating alginates, 96% ethanol and distilled water (Merck (former Sigma-Aldrich), Darmstadt, Germany) were used; sodium alginate from brown algae was used as a control (≥98% purity; Merck (former Sigma-Aldrich), Darmstadt, Germany). Yeast extract (BR) from Macklin, Shanghai, China, Lot no. C14797426, CAS: 8013-01-2, tryptone from Macklin, Shanghai, China, Lot no. C10587432, CAS: 73049-73-7, sodium chloride (≥99.8%, Macklin, Shanghai, China, Lot no. C17929176, CAS: 7647-14-5), calcium chloride (AR, ≥96%, Shanghai Enchem Chemical Technology Co., Ltd., Shanghai, China, Batch no. RH678333, CAS:l0043-52-4), and poly(ethylene glycol) (MW = 8000) used in phage concentration and purification was from Macklin, Shanghai, China, Lot no. C16724290, CAS: 25322-68-3. The DMEM high glucose (JetBiofil, Guangzhou, China, Lot no. 20240622), 10% FBS (Wuhan Pricella Biotechnology Co., Ltd., Wuhan, China, Lot no. SA241025), antibiotic–antimycotic (Gibco, Thermo Fisher Scientific, Waltham, MA, USA)), CCK-8 Cell Viability Assay Kit (Beijing Labgic Technology Co., Ltd., Beijing, China).

### 4.2. Bacterial Alginate Synthesis

*Azotobacter vinelandii* 12 strain, isolated from soddy-podzolic soil located in the Moscow region (Russia), was maintained in the laboratory of biochemistry of nitrogen fixation and nitrogen metabolism of the Bach Institute of Biochemistry of the Federal Research Center “Fundamental Foundations of Biotechnology” of the Russian Academy of Sciences (Moscow, Russia). Bacterial alginate was synthesized and purified according to the method described by Dudun A. et al. [13]. Briefly (Figure S3), *Azotobacter vinelandii* was cultivated in liquid Burke’s medium (chemicals were obtained from Merck, former Sigma-Aldrich, Darmstadt, Germany) with slight modifications as an increase of phosphate concentrations. Capsular alginate was isolated from biomass, then the sample was stirred for 1 h at 60 °C stirring on an orbital shaker PSU-20i (Biosan, Riga, Latvia) until complete homogenization, followed by centrifugation at 11,000 g for 30 min. In order to isolate alginate, three volumes of ethanol cooled to −20 °C were added to the supernatant, and the precipitate was collected, lyophilized, redissolved, dialyzed, precipitated with ethanol, and lyophilized again using Alpha 1-2 LD plus (Martin Christ, Osterode am Harz, Germany).

### 4.3. Bacteriophage Culture and Propagation

The standard laboratory strain of *P. aeruginosa* PAO1 was obtained from the collection of the Laboratory of Bacteriophage Genetics, Mechnikov Research Institute of Vaccines & Sera (Moscow, Russia). Phage phiKZ was kindly donated for the present experimental work by Prof. V. Krylov [48]. PhiKZ was propagated using *P. aeruginosa* PAO1 as the host strain. *P. aeruginosa* was grown overnight in LB broth at 37 °C 200 rpm, diluted to OD600 0.005 with fresh LB, and subsequently infected with phiKZ. Following complete lysis of the bacterial culture, the solution was centrifuged and filtered through a 0.22 μm filter to obtain fresh bacteriophage lysate. For the preparation of high-titer phage stocks (up to 10^12^ PFU/mL) intended for further biomaterial encapsulation, they were concentrated by PEG precipitation according to the protocol by Kunish et al. [49].

### 4.4. Microbead Composition

To produce alginate–chitosan gel microbeads encapsulated with phages, the method proposed by Kaur et al. was modified [8]. All solutions were pre-sterilized by 0.22 μm filtration, except for alginate and chitosan. In the first step, 7.5 mL of 0.65% sterile alginate solution (sterilization by powder autoclaving; seaweed alginate 150 kDa (AppliChem, Darmstadt, Germany) or bacterial alginate 350 kDa [13]) in sodium chloride was added to a glass with a magnetic stirrer at 900 rpm. Then, 500 μL of phage lysate (4 × 10^12^ PFU/mL) or water was added to produce phage-encapsulated microbeads or control empty microbeads. After 2 min of stirring, 1600 μL of CaCl_2_ 0.51 mg/mL was added drop by drop and left for 30 min of stirring to create alginate pre-gel. In the second step, 1600 μL of CaCl_2_ 50 mM or 0.3% chitosan (dissolved from 3% chitosan in 1% acetic acid with 50 mM CaCl_2_) was added dropwise to create the final microbeads. The solutions were stirred for 30 min and then left for equilibration for at least 12 h. In the final step, eight resulting solutions with microbeads were centrifuged and washed with water two times. All microbeads and supernatants were collected for antimicrobial activity testing.

### 4.5. Phage Plaque Assay

Quantification of phage titer in phage release experiments, antimicrobial activity assay, and in supernatants from microbead washing steps was performed by double-layer agar method, as previously described in Bugaeva et al. [50]. Briefly, serial phage lysate’s dilutions (10 μL) were spotted onto LB double-layer agar plates. Following overnight incubation at 37 °C, the plaque-forming units (PFU/mL) were enumerated, according to the Formula (1):

PFU/mL = *N* × *d* × 100, (1)

where *N* represents the numbers of plaques, *d* is the dilution factor.

### 4.6. In Vitro Antimicrobial Activity

Antibacterial activity was evaluated using a modified plate-count assay described by Xu et al. [51]. Briefly, 25 μL of freshly cultured bacteria (adjusted to OD600 = 0.3) was mixed with 250 μL of resuspended microbeads (each microbead type tested separately) in 950 μL of LB broth. Cultivation was performed under shaking conditions of 200 rpm at 37 °C for 3, 6, and 9 h. Bacterial cultures were serially diluted and spread, plated onto LB agar, and incubated for 12 h to determine the CFU. Additionally, for the phage-containing microbeads, an additional aliquot was collected to assess phage release in vitro, and phage titers were quantified as PFU/mL using the double-layer agar method.

### 4.7. Phage Release in Physiological Saline Solution

Phage release was assessed as described previously by Xu et al. [51] with slight modifications. Briefly, the developed microbeads were introduced into physiological saline solution at a ratio of 1:4 in 2 mL tubes, followed by incubation at 37 °C and 200 rpm shaking conditions. At predetermined time intervals (3, 6, 9, 24, and 48 h), the incubation mixture was withdrawn into aliquots and replenished with an equal volume of fresh physiological saline solution. The rate of phage release was quantified using the double-layer plate assay in PFU/mL.

### 4.8. Cytotoxicity

The 3T3 fibroblast cell line (Procell, Wuhan, Hubei, China) was used for performing the cytotoxicity test. Cells were seeded in 96-well plates, 10 000 cells per well, in standard growth media (DMEM High glucose, 10% FBS, 1% anti-anti). Eight types of microbead suspension 200 μL (2 × 10^11^ PFU) and 20 μL phage lysate in 180 μL dH_2_O were incubated in 800 μL of growth media overnight in a shaker at 200 rpm under 37 °C. Microbeads were centrifuged, and the conditioned media were collected for cytotoxicity testing. Fresh media (1 mL) was added to the microbeads and resuspended. When a confluent monolayer was formed by 3T3 cells, the media was changed to conditioned media after centrifugation or microbead suspension in fresh growth media. The cells were incubated for 24 h at 37 °C with 6% CO_2_ or 0.03% CO_2_. Platers were held in an Esco CCL-050T-8-IVF three-gas incubator (Esco, Singapore). Results were obtained with a CCK-8 Cell Viability Assay Kit via Agilent BioTek SH1MF (BioTek, Winooski, VT, USA). All experiments were conducted two times in four repetitions.

### 4.9. Raman Spectroscopy

Microbeads from seaweed alginate (sAN, sAP, sANCS, sAPCS) were freeze-dried for 8 h. The Raman spectra of the dry samples were measured using an inVia Qontor confocal Raman microscope (Renishaw, Wotton-under-Edge, Gloucestershire UK) and a combined Integra-Spectra confocal Raman microscope (NT-MDT, Zelenograd, Russia).

Registration of spectra on an inVia Qontor Renishaw Raman microscope was performed at the exciting wavelength of 785 nm and the following parameters: 1200 L/mm(vis) grating (lines per mm), 50× objective, 5 s exposure time, 140 mW laser power, and a spectral range of 200–1800 nm.

Registration of spectra on an Integra-Spectra microscope was performed using the exciting wavelength of 532 nm, 600 L/mm grating, and 20x objective; the time of spectrum recording was 30 s. Five to seven spectra were recorded from each dried microbead sample on the glass slide.

### 4.10. ATR-FTIR Spectroscopy

IR spectra of microbeads were recorded with a Spectrum Two FT-IR Spectrometer (Perkin-Elmer, Waltham, MA, USA) combined with an attenuated total reflectance (ATR) accessory made of Zinc Selene (ZnSe). Glass slides with dried microbeads were pressed to the top plate and then fitted with a ZnSe crystal to ensure good contact between the sample and the crystal. The FTIR absorbance spectra was recorded from 550 to 4000 cm^−1^ with a resolution of 4 cm^−1^ and four scan accumulations. For each sample, the spectra in three different points of the sample were recorded and averaged. The baseline for Raman and FTIR spectra was subtracted using the Spectragryph 1.2 software.

### 4.11. SEM

The microbeads based on seaweed alginate were dialyzed against dH_2_O and air dried on glass, attached to a metal substrate using the carbon double-sided tape, and sputter-coated for 40 s two times in Ar atmosphere with gold in an SBC-12 ion sputter coater (KYKY, Beijing, China) apparatus. SEM images were obtained in a scanning electron microscope EM6200 (KYKY, Beijing, China) at ×10 K magnification.

### 4.12. Zeta-Sizer

The hydrodynamic diameters of eight types of microbeads were determined by dynamic light scattering (DLS) in physiological saline on a Zetasizer Nano ZS particle analyzer (Malvern Instruments, Malvern, Worcestershire, UK). The ζ-potentials of microbeads based on seaweed alginate were measured in physiological saline on a Litesizer DLS 700 (Anton Paar, Graz, Styria, Austria). Disposable plastic cuvettes were used. The average values of a minimum of ten runs before and after mixing were obtained. The data was processed using Excel and GraphPad Prism tools.

### 4.13. Statistical Analysis

The number of replicates and sample sizes are indicated in the corresponding tables, figures, and figure legends. All experiments were performed in at least triplicate unless otherwise stated, and results are presented as mean ± standard deviation (SD). Statistical analyses were performed using GraphPad Prism version 10.6.0 (GraphPad Software, San Diego, CA, USA).

Comparisons between groups were performed using unpaired two-tailed Student’s *t*-test, unpaired two-tailed Welch’s *t*-test, or one-way ANOVA followed by Tukey’s post hoc multiple comparison test, as appropriate. A *p*-value < 0.05 was considered statistically significant. The statistical methods and exact *p*-values or significance levels are indicated in the corresponding figures and figure legends.

For Raman and FTIR spectra processing, we used the free spectroscopy software SpectraGryph 1.2 (spectragryph.com) for spectra averaging and baseline subtraction, and we used a demo version of Origin, Version 2022, (OriginLab Corporation, Northampton, MA, USA) to determine peak positions.

## Figures and Tables

**Table 1:** Microbead composition.

Code	Alginate Type	ALG 0.065%	Phage 4 × 10^12^ PFU/mL	SM Buffer	CaCl_2_ 50 mM	Chitosan 0.3% in CaCl_2_ 50 mM
bAN	bacterial	7.5 mL	-	500 μL	1600 μL	-
bAP	bacterial	7.5 mL	500 μL	-	1600 μL	-
bANCS	bacterial	7.5 mL	-	500 μL	-	1600 μL
bAPCS	bacterial	7.5 mL	500 μL	-	-	1600 μL
sAN	seaweed	7.5 mL	-	500 μL	1600 μL	-
sAP	seaweed	7.5 mL	500 μL	-	1600 μL	-
sANCS	seaweed	7.5 mL	-	500 μL	-	1600 μL
sAPCS	seaweed	7.5 mL	500 μL	-	-	1600 μL

**Table 2:** Phage titer load during microbead production steps.

	sAP	sAPCS	bAP	bAPCS
Initial load (PFU/mL)	4.3 × 10^12^	4.3 × 10^12^	4.3 × 10^12^	4.3 × 10^12^
Losses in wash step 1 (PFU/mL)	1 × 10^11^	1.5 × 10^6^	9 × 10^10^	2.2 × 10^8^
Losses in wash step 2 (PFU/mL)	8 × 10^10^	6 × 10^5^	6.2 × 10^9^	3 × 10^7^
Final load (PFU/mL)	8.4 × 10^11^	1.1 × 10^12^	1.2 × 10^12^	1.1 × 10^12^
Final load (PFU/mg dry mass)	2.1 × 10^14^	1.4 × 10^14^	2.9 × 10^14^	1.4 × 10^14^

**Table 3:** ζ-potential and conductivity of microbead samples.

Sample	Mean ζ-Potential * ± SD, mV	Conductivity **, mS/cm
sAN ***	−36.6 ± 3.3	12.88
sAP	−33.1 ± 2.7	12.56
sANCS	+4.3 ± 1.5	14.44
sAPCS	−18.7 ± 1.3	11.89

* Zeta-potential values are reported as mean ± SD obtained from 50 instrument reads (*n* = 2). ** Conductivity was recorded as a single instrument-measured value during each measurement cycle. *** Alginate-based beads without and with phage—sAN and sAP, alginate–chitosan beads without and with phage—sANCS and sAPCS (according to Table 1).

## Data Availability

The original data presented in the study are openly available in Zenodo at https://doi.org/10.5281/zenodo.20521728.

## Supplementary Materials

The following supporting information can be downloaded at: https://www.mdpi.com/article/10.3390/gels12060544/s1, Figure S1. Representative photographs of Petri dishes displaying *Pseudomonas aeruginosa* colonies following treatment with seaweed alginate microbeads or in untreated control samples; Figure S2. Microbeads (a) and their supernatant after 24 h incubation (b) cytotoxicity under low CO_2_ condition (*n* = 10, one-way ANOVA); Figure S3. Scheme of bacterial alginate synthesis (described by Dudun A. et al. [13]); Table S1. Quantitative assessment of *P. aeruginosa* CFU/mL counts following treatment with microbeads based on seaweed alginate and bacterial alginate formulations (represented as mean value, *n* = 3).

## Abbreviations

The following abbreviations are used in this manuscript:

bALG	alginate of bacterial origin
sALG	alginate of seaweed origin
bAN	blank bacterial alginate microbeads
bANCS	blank bacterial chitosan–alginate microbeads
bAP	phage-loaded bacterial alginate microbeads
bAPCS	phage-loaded bacterial chitosan–alginate microbeads
sAN	blank seaweed alginate microbeads
sANCS	blank seaweed chitosan–alginate microbeads
sAP	phage-loaded seaweed alginate microbeads
sAPCS	phage-loaded seaweed chitosan–alginate microbeads
ATR	attenuated total reflectance
CFU	colony-forming units
PDI	polydispersity index
PFU	plaque-forming units

## References

[1] Shen T., Dai K., Zhang S., Wang J., Liu C. (2025). Injured Bone-Triggered Osteokines Secretion Promotes Diabetic Wound Healing. Bone Res..

[2] Hofbauer L.C., Busse B., Eastell R., Ferrari S., Frost M., Müller R., Burden A.M., Rivadeneira F., Napoli N., Rauner M. (2022). Bone Fragility in Diabetes: Novel Concepts and Clinical Implications. Lancet Diabetes Endocrinol..

[3] Naghavi M., Vollset S.E., Ikuta K.S., Swetschinski L.R., Gray A.P., Wool E.E., Robles Aguilar G., Mestrovic T., Smith G., Han C. (2024). Global Burden of Bacterial Antimicrobial Resistance 1990–2021: A Systematic Analysis with Forecasts to 2050. Lancet.

[4] Murray C.J.L., Ikuta K.S., Sharara F., Swetschinski L., Robles Aguilar G., Gray A., Han C., Bisignano C., Rao P., Wool E. (2022). Global Burden of Bacterial Antimicrobial Resistance in 2019: A Systematic Analysis. Lancet.

[5] Zhang C., Fu X., Liu Y., Zhao H., Wang G. (2024). Burden of Infectious Diseases and Bacterial Antimicrobial Resistance in China: A Systematic Analysis for the Global Burden of Disease Study 2019. Lancet Reg. Heal. West. Pac..

[6] Ahmad N., Bukhari S.N.A., Hussain M.A., Ejaz H., Munir M.U., Amjad M.W. (2024). Nanoparticles Incorporated Hydrogels for Delivery of Antimicrobial Agents: Developments and Trends. RSC Adv..

[7] Nguyen N.H., Lu Z., Elbourne A., Vasilev K., Roohani I., Zreiqat H., Truong V.K. (2024). Engineering Antibacterial Bioceramics: Design Principles and Mechanisms of Action. Mater. Today Bio.

[8] Kaur J., Kour A., Panda J.J., Harjai K., Chhibber S. (2020). Exploring Endolysin-Loaded Alginate-Chitosan Nanoparticles as Future Remedy for Staphylococcal Infections. AAPS PharmSciTech.

[9] Satchanska G., Davidova S., Petrov P.D. (2024). Natural and Synthetic Polymers for Biomedical and Environmental Applications. Polymers.

[10] Baysal K., Aroguz A.Z., Adiguzel Z., Baysal B.M. (2013). Chitosan/Alginate Crosslinked Hydrogels: Preparation, Characterization and Application for Cell Growth Purposes. Int. J. Biol. Macromol..

[11] Yan D., Li Y., Liu Y., Li N., Zhang X., Yan C. (2021). Antimicrobial Properties of Chitosan and Chitosan Derivatives in the Treatment of Enteric Infections. Molecules.

[12] Aderibigbe B., Buyana B. (2018). Alginate in Wound Dressings. Pharmaceutics.

[13] Dudun A., Akoulina E., Zhuikov V., Makhina T., Voinova V., Belishev N., Khaydapova D., Shaitan K., Bonartseva G., Bonartsev A. (2021). Competitive Biosynthesis of Bacterial Alginate Using *Azotobacter vinelandii* 12 for Tissue Engineering Applications. Polymers.

[14] Chung J., Eisha S., Park S., Morris A.J., Martin I. (2023). How Three Self-Secreted Biofilm Exopolysaccharides of *Pseudomonas aeruginosa*, Psl, Pel, and Alginate, Can Each Be Exploited for Antibiotic Adjuvant Effects in Cystic Fibrosis Lung Infection. Int. J. Mol. Sci..

[15] Vasina D.V., Antonova N.P., Shidlovskaya E.V., Kuznetsova N.A., Grishin A.V., Akoulina E.A., Trusova E.A., Lendel A.M., Mazunina E.P., Kozlova S.R. (2024). Alginate Gel Encapsulated with Enzybiotics Cocktail Is Effective against Multispecies Biofilms. Gels.

[16] Zhang B., Wang Y., Wang F., Zhang Y., Hao H., Lv X., Hao L., Shi Y. (2023). Microencapsulated Phage Composites with Increased Gastrointestinal Stability for the Oral Treatment of *Salmonella* Colonization in Chicken. Front. Vet. Sci..

[17] Pinto A.M., Cerqueira M.A., Bañobre-Lópes M., Pastrana L.M., Sillankorva S. (2020). Bacteriophages for Chronic Wound Treatment: From Traditional to Novel Delivery Systems. Viruses.

[18] Moghtader F., Solakoglu S., Piskin E. (2024). Alginate- and Chitosan-Modified Gelatin Hydrogel Microbeads for Delivery of *E. coli* Phages. Gels.

[19] Chen B., Benavente L.P., Chittò M., Wychowaniec J.K., Post V., D’Este M., Constant C., Zeiter S., Feng W., Moreno M.G. (2023). Alginate Microbeads and Hydrogels Delivering Meropenem and Bacteriophages to Treat *Pseudomonas aeruginosa* Fracture-Related Infections. J. Control. Release.

[20] Abdelsattar A.S., Abdelrahman F., Dawoud A., Connerton I.F., El-Shibiny A. (2019). Encapsulation of *E. coli* Phage ZCEC5 in Chitosan–Alginate Beads as a Delivery System in Phage Therapy. AMB Express.

[21] Rotman S.G., Sumrall E., Ziadlou R., Grijpma D.W., Richards R.G., Eglin D., Moriarty T.F. (2020). Local Bacteriophage Delivery for Treatment and Prevention of Bacterial Infections. Front. Microbiol..

[22] Rotman S.G., Post V., Foster A.L., Lavigne R., Wagemans J., Trampuz A., Moreno M.G., Metsemakers W.-J., Grijpma D.W., Richards R.G. (2023). Alginate Chitosan Microbeads and Thermos-Responsive Hyaluronic Acid Hydrogel for Phage Delivery. J. Drug Deliv. Sci. Technol..

[23] Drury J.L., Dennis R.G., Mooney D.J. (2004). The Tensile Properties of Alginate Hydrogels. Biomaterials.

[24] dos Santos Silva M., Cocenza D.S., Grillo R., de Melo N.F.S., Tonello P.S., de Oliveira L.C., Cassimiro D.L., Rosa A.H., Fraceto L.F. (2011). Paraquat-Loaded Alginate/Chitosan Nanoparticles: Preparation, Characterization and Soil Sorption Studies. J. Hazard. Mater..

[25] Dehari D., Chaudhuri A., Kumar D.N., Patil R., Gangwar M., Rastogi S., Kumar D., Nath G., Agrawal A.K. (2023). A Bacteriophage Microgel Effectively Treats the Multidrug-Resistant *Acinetobacter baumannii* Bacterial Infections in Burn Wounds. Pharmaceuticals.

[26] Yilmaz T., Maldonado L., Turasan H., Kokini J. (2019). Thermodynamic Mechanism of Particulation of Sodium Alginate and Chitosan Polyelectrolyte Complexes as a Function of Charge Ratio and Order of Addition. J. Food Eng..

[27] Atma Y., Sadeghpour A., Murray B.S., Goycoolea F.M. (2025). Chitosan-Alginate Polyelectrolyte Complexes for Encapsulation of Low Molecular Weight Fish Bioactive Peptides. Food Hydrocoll..

[28] Ibrahim H.K., Sorour R.M.H., Salah Ad-Din I. (2022). Application of Mathematical Modelling to Alginate Chitosan Polyelectrolyte Complexes for the Prediction of System Behavior with Venlafaxine HCl as a Model Charged Drug. Saudi Pharm. J..

[29] Yeerong K., Chantawannakul P., Anuchapreeda S., Juntrapirom S., Kanjanakawinkul W., Müllertz A., Rades T., Chaiyana W. (2024). Chitosan Alginate Nanoparticles of Protein Hydrolysate from Acheta Domesticus with Enhanced Stability for Skin Delivery. Pharmaceutics.

[30] Hu C., Lu W., Mata A., Nishinari K., Fang Y. (2021). Ions-Induced Gelation of Alginate: Mechanisms and Applications. Int. J. Biol. Macromol..

[31] Michen B., Graule T. (2010). Isoelectric Points of Viruses. J. Appl. Microbiol..

[32] Ly-Chatain M.H., Moussaoui S., Vera A., Rigobello V., Demarigny Y. (2013). Antiviral Effect of Cationic Compounds on Bacteriophages. Front. Microbiol..

[33] Sikora M., Wąsik S., Semaniak J., Drulis-Kawa Z., Wiśniewska-Wrona M., Arabski M. (2024). Chitosan-Based Matrix as a Carrier for Bacteriophages. Appl. Microbiol. Biotechnol..

[34] Zhou Y., Franks G.V. (2006). Flocculation Mechanism Induced by Cationic Polymers Investigated by Light Scattering. Langmuir.

[35] Lomeli-Martin A., Azad Z., Thomas J.A., Lapizco-Encinas B.H. (2024). Assessment of the Nonlinear Electrophoretic Migration of Nanoparticles and Bacteriophages. Micromachines.

[36] Feraru A., Tóth Z.-R., Mureșan-Pop M., Baia M., Gyulavári T., Páll E., Turcu R.V.F., Magyari K., Baia L. (2023). Anionic Polysaccharide Cryogels: Interaction and In Vitro Behavior of Alginate–Gum Arabic Composites. Polymers.

[37] Schmid T., Messmer A., Yeo B.-S., Zhang W., Zenobi R. (2008). Towards Chemical Analysis of Nanostructures in Biofilms II: Tip-Enhanced Raman Spectroscopy of Alginates. Anal. Bioanal. Chem..

[38] Zając A., Hanuza J., Wandas M., Dymińska L. (2015). Determination of N-Acetylation Degree in Chitosan Using Raman Spectroscopy. Spectrochim. Acta Part A Mol. Biomol. Spectrosc..

[39] Rehman H.U., Cord-Landwehr S., Shapaval V., Dzurendova S., Kohler A., Moerschbacher B.M., Zimmermann B. (2023). High-Throughput Vibrational Spectroscopy Methods for Determination of Degree of Acetylation for Chitin and Chitosan. Carbohydr. Polym..

[40] Gieroba B., Sroka-Bartnicka A., Kazimierczak P., Kalisz G., Lewalska-Graczyk A., Vivcharenko V., Nowakowski R., Pieta I.S., Przekora A. (2022). Surface Chemical and Morphological Analysis of Chitosan/1,3-β-d-Glucan Polysaccharide Films Cross-Linked at 90 °C. Int. J. Mol. Sci..

[41] Ryan C.C., Bardosova M., Pemble M.E. (2017). Structural and Mechanical Properties of a Range of Chitosan-Based Hybrid Networks Loaded with Colloidal Silica and Polystyrene Particles. J. Mater. Sci..

[42] Kim S., Jo A., Ahn J. (2015). Application of Chitosan–Alginate Microspheres for the Sustained Release of Bacteriophage in Simulated Gastrointestinal Conditions. Int. J. Food Sci. Technol..

[43] Temsaah H.R., Abdelkader K., Ahmed A.E., Elgiddawy N., Eldin Z.E., Elshebrawy H.A., Kasem N.G., El-Gohary F.A., Azmy A.F. (2025). Chitosan Nano-Formulation Enhances Stability and Bactericidal Activity of the Lytic Phage HK6. BMC Biotechnol..

[44] Gomaa I., Emam M.H., Wassel A.R., Ashraf K., Hussan S., Kalil H., Bayachou M., Ibrahim M.A. (2023). Microspheres with 2D RGO/Alginate Matrix for Unusual Prolonged Release of Cefotaxime. Nanomaterials.

[45] Akoulina E., Dudun A., Bonartsev A., Bonartseva G., Voinova V. (2019). Effect of Bacterial Alginate on Growth of Mesenchymal Stem Cells. Int. J. Polym. Mater. Polym. Biomater..

[46] Dehari D., Kumar D.N., Chaudhuri A., Kumar A., Kumar R., Kumar D., Singh S., Nath G., Agrawal A.K. (2023). Bacteriophage Entrapped Chitosan Microgel for the Treatment of Biofilm-Mediated Polybacterial Infection in Burn Wounds. Int. J. Biol. Macromol..

[47] Shen H.-Y., Liu Z.-H., Hong J.-S., Wu M.-S., Shiue S.-J., Lin H.-Y. (2021). Controlled-Release of Free Bacteriophage Nanoparticles from 3D-Plotted Hydrogel Fibrous Structure as Potential Antibacterial Wound Dressing. J. Control. Release.

[48] Krylov V.N., Zhazykov I.Z. (1978). Pseudomonas Bacteriophage PhiKZ—A Possible Model for Studying the Genetic Control of Mor-Phogenesis. Genetika.

[49] Kunisch F., Wagemans J., Moreno M.G. (2023). Bacteriophage Precipitation with Polyethylene Glycol (PEG). Protoc. Exch..

[50] Bugaeva E.N., Voronina M.V., Vasiliev D.M., Lukianova A.A., Landyshev N.N., Ignatov A.N., Miroshnikov K.A. (2021). Use of a Specific Phage Cocktail for Soft Rot Control on Ware Potatoes: A Case Study. Viruses.

[51] Xu Y., Yang T., Miao Y., Zhang Q., Yang M., Mao C. (2024). Injectable Phage-Loaded Microparticles Effectively Release Phages to Kill Methicillin-Resistant *Staphylococcus aureus*. ACS Appl. Mater. Interfaces.

