# Supplementary material for: Alginate–Chitosan Gel Microbeads for PhiKZ Encapsulation as a Model of Bacteriophage Delivery to Combat Pseudomonas aeruginosa
Source: Gels. 2026 Jun 17;12(6):544. doi: 10.3390/gels12060544 (PMC13298530; doi:10.3390/gels12060544)
Supplement: Supplementary file 1 [file gels-12-00544-s001.zip › gels-4314239-supplementary.pdf]

Supplementary materials

Alginate–Chitosan Gel Microbeads for PhiKZ Encapsulation as a Model of Bacteriophage Delivery to Combat *Pseudomonas aeruginosa*

Liubov I. Popova [1,†], Elizaveta A. Akoulina [1,2,*,†], Evgeniia Yu. Parshina [2], Timofey A. Tarasov [1], Hejia Yue [1], Qing Peng [3], Ying Zhang [3], Andrei A. Dudun [4], Anton P. Bonartsev [2], Olga S. Sokolova [1,2] and Tolbert Osire [1,*]

Table S1. Quantitative assessment of *P. aeruginosa* CFU/ml counts following treatment with microbeads based on seaweed alginate and bacterial alginate formulations (represented as mean value, n=3).

Alginate source	Sample/incubation time	3h	6h	9h
-	control (*P. aeruginosa only*)	1.15×10^{11}	8.83×10^{11}	9.7×10^{12}
seaweed	sAN	2.09×10^{11}	6.37×10^{12}	4.56×10^{12}
	sAP	3.23×10^{4}	1.29×10^{5}	6.8×10^{6}
	sANCS	1.5×10^{11}	7.23×10^{11}	3.6×10^{12}
	sAPCS	8.43×10^{6}	1.05×10^{6}	1.98×10^{7}
bacterial	bAN	8.57×10^{10}	2.73×10^{12}	5.72×10^{12}
	bAP	1.93×10^{5}	2.12×10^{6}	3.04×10^{8}
	bANCS	6.83×10^{10}	1.38×10^{13}	7.94×10^{12}
	bAPCS	7.03×10^{6}	2.29×10^{6}	2.41×10^{7}

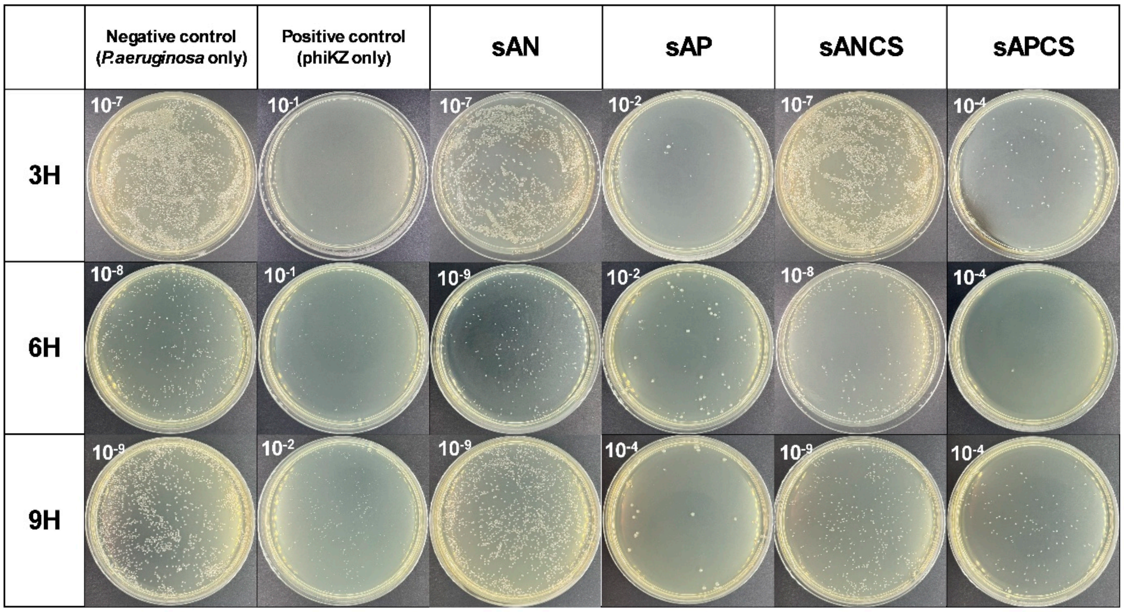

Figure S1. Representative photographs of Petri dishes displaying *Pseudomonas aeruginosa* colonies following treatment with seaweed alginate microbeads or in untreated control samples.

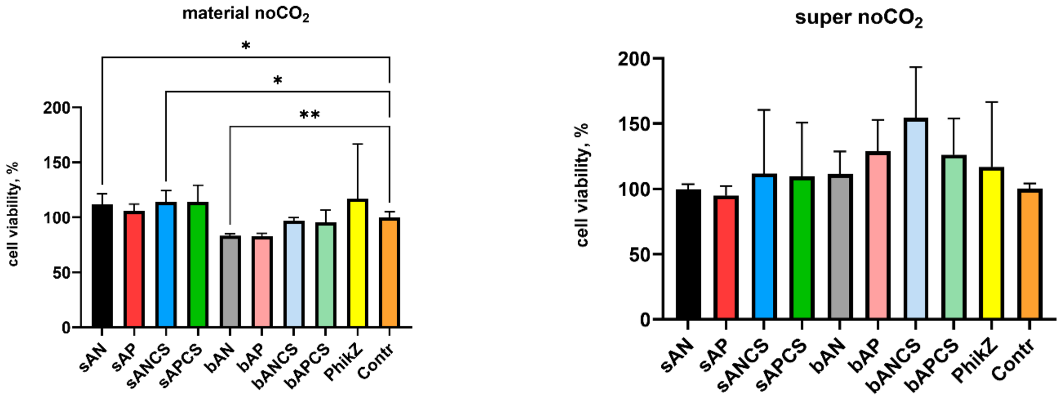

(a) (b)

Figure S2. Microbeads (a) and their supernatant after 24 hours incubation (b) cytotoxicity under low CO2 condition (n=10, one-way ANOVA).

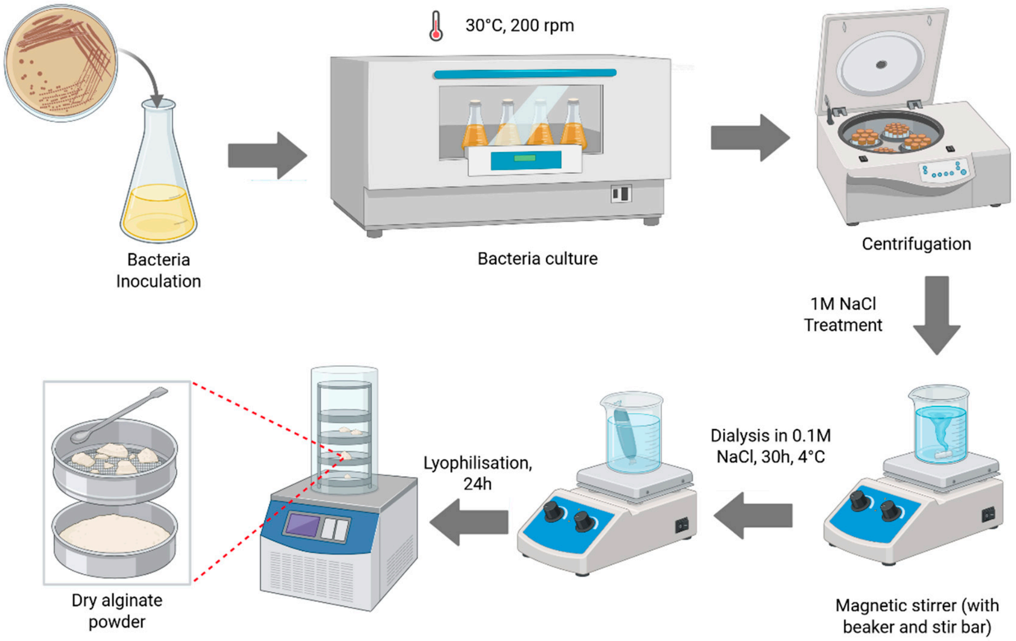

Figure S3. Scheme of bacterial alginate synthesis (described by Dudun A. et al [13]).
